# Characterization of the biosynthetic gene cluster of the polyene macrolide antibiotic reedsmycins from a marine-derived *Streptomyces* strain

**DOI:** 10.1186/s12934-018-0943-6

**Published:** 2018-06-19

**Authors:** Tingting Yao, Zengzhi Liu, Tong Li, Hui Zhang, Jing Liu, Huayue Li, Qian Che, Tianjiao Zhu, Dehai Li, Wenli Li

**Affiliations:** 1 Key Laboratory of Marine Drugs, Ministry of Education of China, School of Medicine and Pharmacy, Ocean University of China, Qingdao, 266003 China; 2 Laboratory for Marine Drugs and Bioproducts of Qingdao National Laboratory for Marine Science and Technology, Qingdao, 266237 China

**Keywords:** Gene cluster, Polyene macrolide, Biosynthesis, Reedsmycins, Marine-derived *Streptomyces* strain

## Abstract

**Background:**

Polyene antibiotics are important as antifungal medicines albeit with serious side effects such as nephrotoxicity. Reedsmycin (RDM) A (**1**), produced by marine-derived *Streptomyces youssoufiensis* OUC6819, is a non-glycosylated polyene macrolide antibiotic with antifungal activity comparable to that of clinically used nystatin. To elucidate its biosynthetic machinery, herein, the *rdm* biosynthetic gene cluster was cloned and characterized.

**Results:**

The *rdm* cluster is located within a 104 kb DNA region harboring 21 open reading frames (ORFs), among which 15 ORFs were designated as *rdm* genes. The assembly line for RDM A is proposed on the basis of module and domain analysis of the polyketide synthetases (PKSs) RdmGHIJ, which catalyze 16 rounds of decarboxylative condensation using malonyl-CoA as the starter unit (loading module), two methylmalonyl-CoA (module 1 and 2), and fourteen malonyl-CoA (module 3–16) as extender units successively. However, the predicted substrate specificity of AT0 in the loading module is methylmalonyl-CoA instead of malonyl-CoA. Interestingly, the *rdm* cluster contains a five-gene regulation system RdmACDEF, which is different from other reported polyene gene clusters. In vivo experiments demonstrated the XRE family regulator RdmA and the PAS/LuxR family regulator RdmF function in negative and positive manner, respectively. Notably, inactivation of *rdmA* and overexpression of *rdmF* led to increased production of RDM A by ~ 2.0-fold and ~ 2.5-fold, reaching yields of 155.3 ± 1.89 and 184.8 ± 9.93 mg/L, respectively.

**Conclusions:**

Biosynthesis of RDM A is accomplished on a linear assembly line catalyzed by Rdm PKSs harboring a unique AT0 under the control of a complex regulatory system. These findings enable generation of new biologically active RDM derivatives at high yield and with improved properties by engineered biosynthesis.

**Electronic supplementary material:**

The online version of this article (10.1186/s12934-018-0943-6) contains supplementary material, which is available to authorized users.

## Background

Pathogenic fungi are a leading cause of human mortality, particularly among an ever-increasing population of immunocompromised individuals, and have become critical threats to global health [1]. Polyenes were the first antifungal drugs for clinical use. Compared to any other antifungal agents, they have the broadest spectrum of activity [1]. Up to date, polyene macrolide antibiotics have been proved to be one of the most effective antifungal agents, especially when dealing with life-threatening systemic fungal infections [2]. However, the emergence of rare or unidentified species of drug-resistant fungal pathogens and the clinical need for non-toxic antifungal drugs demand us to develop new polyene derivatives and formulations, given the apparent toxicity and serious side effects of existing polyene macrolides [2, 3].

The biosynthetic mechanisms for several polyene macrolide antibiotics, including candicidin (FR-008) [4, 5], amphotericin [6], pimaricin [7], nystatin [8], tetramycin [9], and NPP [10] have been widely studied. The formation of the macrolactone ring is typically catalyzed by a modular type I polyketide synthetase (PKS), after which two tailoring steps usually occur to furnish the exocyclic carboxyl group and the mycosamine sugar(s) [11]. Engineering of the polyene PKSs and tailoring genes have generated several polyene analogues. For example, inactivation of KR16 in amphotericin PKS resulted in 7-*oxo*-amphotericin B, which had good antifungal activity and was less hemolytic than amphotericin B [12]. By inactivation of the corresponding P450 gene in each cluster, the exocyclic carboxyl group was replaced with a methyl group in amphotericin B [13], nystatin [14], candicidin [15], and rimocidin [16], leading to decreased haemolytic activity but not antifungal activity. Recently, Kim et al. [17] carried out cross genetic complementation of the *Pseudonocardia autotrophica* ∆*nppY* mutant strain with *nypY* from *Pseudonocardia* sp. P1, leading to isolation of a mannosylated NPP analogue with reduced antifungal activity while higher nephrotoxical activity against human hepatocytes. Albeit many potentially valuable polyene analogues have been developed, none of them has advanced into clinical medicine so far [11].

**Fig. 1:**
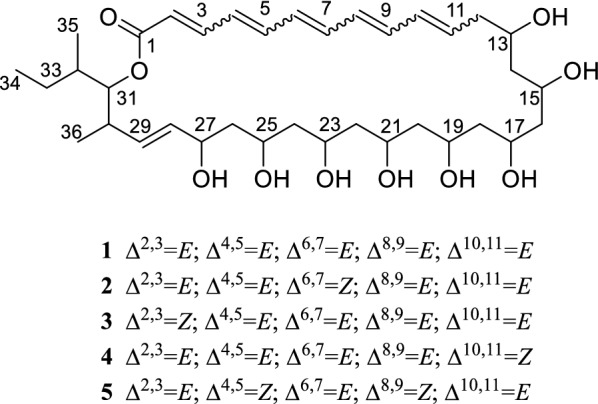
Chemical structures of reedsmycins (RDMs, **1**–**5**). RDM A (**1**) is the major component

Genome sequencing has uncovered an increasing number of polyene biosynthetic gene clusters, facilitating genome-directed novel polyene discovery [11]. Previously, we did genome scanning of the reeds rhizosphere soil-derived *Streptomyces youssoufiensis* OUC6819 (previously *Streptomyces* sp. CHQ-64) and found a 28-kb DNA fragment encoding two partial type I PKSs [18]. Bioinformatic analysis indicated it is comprised of 6 modules putatively encoding a polyene-polyol compound. Inspired by this fact, we identified a series of reedsmycins (RDMs **1**–**5**, Fig. 1), featuring a polyene-polyol macrolide devoid of sugar moiety. Notably, the major component reedsmycin A (**1**) (RDM A) exhibited comparable inhibition against *Candida albicans* as that of the positive control nystatin [18]. The purpose of this study was to verify the involvement of this 28-kb DNA fragment in the biosynthesis of RDMs, and characterize the entire *rdm* gene cluster to set the stage to genetically engineer new bioactive RDM analogues at high yield. Herein we report (i) the cloning and sequencing of the *rdm* gene cluster; (ii) bioinformatics analysis of the *rdm* cluster and a proposed assembly line of RDM A; (iii) gene inactivation to support function of the *rdm* cluster; (iv) genetic characterization of the unique regulatory system for RDM biosynthesis.

## Results

### Cloning and sequencing of the entire *rdm* cluster

**Fig. 2:**
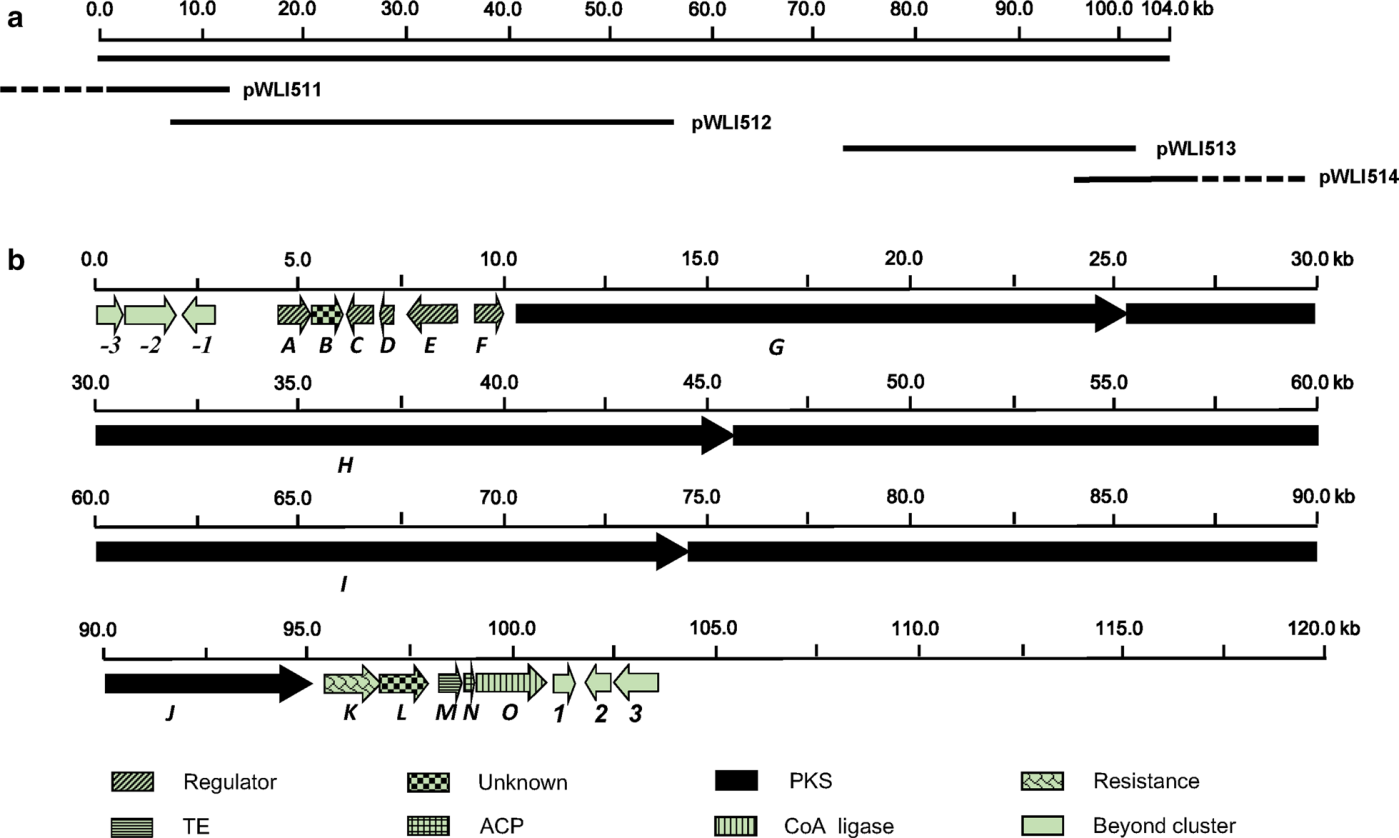
The DNA region harboring the RDM biosynthetic gene cluster (**a**) and its genetic organization (**b**). Four cosmids harboring the *rdm* genes were indicated. Proposed functions of individual ORFs are coded with various patterns and summarized in Table 1

The cosmid library of *S. youssoufiensis* OUC6819 was firstly constructed and screened by PCR, resulting in four positive cosmids pWLI511–514 (Fig. 2a). The sequence gaps within the PKS genes were filled by chromosomal walking using the pWLI512 or pWLI513 as template. Combined with our previous genome scanning results, a 104-kb DNA sequence containing the entire *rdm* cluster was obtained and deposited in GenBank database under the accession number MG947597. Twenty-one open reading frames (ORFs) were identified within the sequence (Fig. 2b), and their proposed functions were summarized in Table 1, among which fifteen ORFs were designated as *rdm* genes. The deduced encoding products of the *rdm* genes include four PKSs (RdmG, RdmH, RdmI, and RdmJ), five regulatory proteins (RdmA, RdmC, RdmD, RdmE and RdmF), one resistance protein RdmK, one PaaI family thioesterase (TE) RdmM, one acyl carrier protein (ACP) RdmN, one acyl-CoA synthetase RdmO, and two proteins with predicted functions of methyltransferase (RdmB) and ferritin (RdmL) (Table 1).

**Table 1:** Proposed functions of proteins encoded by the *rdm* biosynthetic gene cluster in *S. youssoufiensis* OUC6819

Protein	Size (aa)	Proposed function	Homologs	
			Protein/organism	Accession no. (identity/similarity %)
Orf(−3)	213	TetR/AcrR family transcriptional regulator	IF52_RS0105620/*Streptomyces ruber*	WP_030357119.1 (66/74)
Orf(−2)	418	FAD-binding monooxygenase	BCAV_RS14010/*Beutenbergia cavernae*	WP_015883264.1 (59/69)
Orf(−1)	267	Methyltransferase	H299_RS35255/*Streptomyces* sp. CNH287	WP_051262671.1 (68/80)
RdmA	286	XRE family transcriptional regulator	ASD26_RS06115/*Streptomyces* sp. Root1319	WP_056788122.1 (57/65)
RdmB	256	Methyltransferase	AB852_RS08905/*Streptomyces uncialis*	WP_073785832.1 (63/73)
RdmC	218	LuxR family two-component system response regulator	KasW*/Streptomyces kasugaensis*	BAF79686.1 (84/91)
RdmD	111	Two-component system sensor kinase	KasX*/S. kasugaensis*	BAF79687.1 (66/81, the 475th–585th aa)
RdmE	410	Two-component system sensor kinase	KasX*/S. kasugaensis*	BAF79687.1 (68/75, the 31st–413rd aa)
RdmF	233	PAS-LuxR regulator	AOM46_RS26735/*Streptomyces* sp. NBRC 109706	WP_062215992.1 (83/89)
RdmG	4996	Type I polyketide synthase	AOM46_RS26740/*Streptomyces* sp. NBRC 109706	WP_078857234.1 (70/78)
RdmH	6440	Type I polyketide synthase	AOM46_RS26745/*Streptomyces* sp. NBRC 109706	WP_078857210.1 (71/80)
RdmI	9533	Type I polyketide synthase	SHXM_01039/*Streptomyces hygroscopicus* XM201	AQW47576.1 (52/63)
RdmJ	7252	Type I polyketide synthase	AOM46_RS26755/*Streptomyces* sp. NBRC 109706	WP_062216000.1 (74/82)
RdmK	498	MFS transporter	AOK24_RS06280/*Streptomyces niveiscabiei*	WP_055719056.1 (76/83)
RdmL	398	Ferritin	AOK24_RS06275/*S. niveiscabiei*	WP_055719055.1 (84/91)
RdmM	172	PaaI family thioesterase	AOK24_RS06270/*S. niveiscabiei*	WP_055719054.1 (75/85)
RdmN	82	Acyl carrier protein	AOK24_RS06265/*S. niveiscabiei*	WP_055719053.1 (54/75)
RdmO	576	Acyl-CoA synthetase	AOK24_RS06260/*S. niveiscabiei*	WP_055719052.1 (66/76)
Orf1	176	Hypothetical protein	AMJ94_18775/*Deltaproteobacteria bacterium* SM23_61 AOK24_RS06255/*S. niveiscabiei* AOK24_RS06250/*S. niveiscabiei*	KPK85730.1 (32/53) WP055719051.1 (78/84, the 1st–79th aa) WP055719050.1 (82/89, the 81st–174th aa)
Orf2	207	DNA-binding response regulator	AOK24_RS06245/*S. niveiscabiei*	WP_063799404.1 (61/74)
Orf3	361	Hypothetical protein	AOK24_RS06240/*S. niveiscabiei*	WP_079056694.1 (56/67)

### Genes encoding modular PKSs

**Fig. 3:**
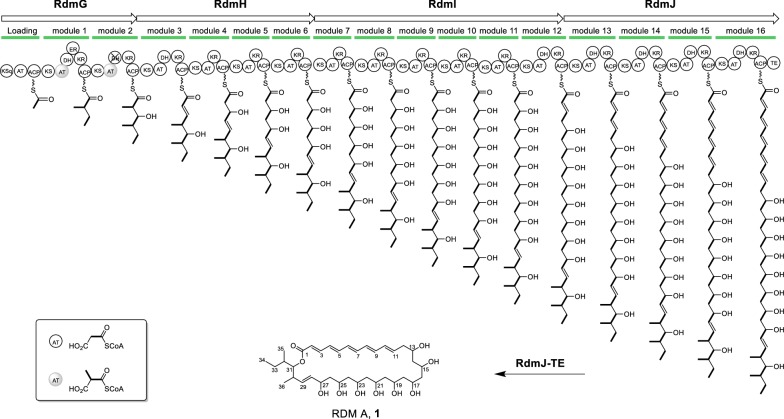
Deduced module and domain organization of RdmGHIJ and a proposed biosynthetic pathway of RDM A (**1**). The AT domains are coded with various patterns to highlight their substrate specificity; “X” indicates domain whose activity appears to be unnecessary. *AT* acyl transferase, *ACP* acyl carrier protein, *KS* ketosynthase, *DH* dehydratase, *KR* ketoreductase, *ER* enoylreductase, *TE* thioesterase

Four large type I PKSs were identified within the *rdm* cluster. *rdmG* encodes the loading module and extension modules 1–2; *rdmH* encodes extension modules 3–6; and *rdmI* encodes extension modules 7–12; and *rdmJ* encodes extension modules 13-–16 and a C-terminal TE domain (Fig. 3). Our previously reported 28-kb fragment covers modules 9–14 [18]. Altogether, RdmG–J is composed of one loading module and sixteen extension modules, consistent with the 34-carbon skeleton of RDMs (Fig. 1).

**Fig. 4:**
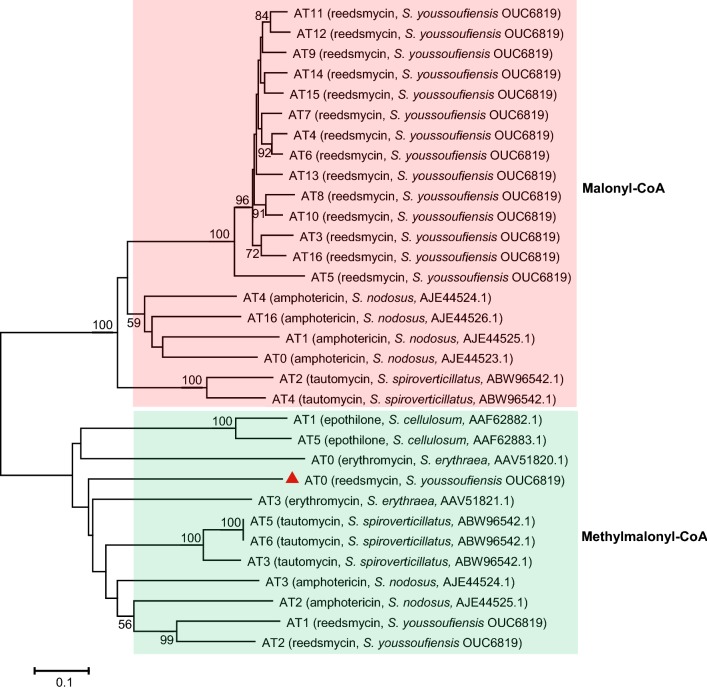
Phylogenetic analysis of RdmGHIJ AT domains with selected characterized ATs from type I PKSs. The percentage of replicate trees in which the associated taxa clustered together in the bootstrap test (1000 replicates) is shown next to the branches. The sequences subjected to alignment are from amphotericin gene cluster in *Streptomyces nodosus*, tautomycin gene cluster in *Streptomyces spiroverticillatus*, epothilone gene cluster in *Sorangium cellulosum*, and erythromycin gene cluster in *Saccharopolyspora erythraea.* Cluster names, producer strains and accession numbers for each of the AT domains are given in parentheses. The scale bar represents 0.1 amino acid substitution per position

The functions of all the domains were deduced based on sequence homology. Except the loading module, which contains a mutated ketosynthase (KSq) with Cys replaced by Gln, the other KS domains contain the CHH/N catalytic triad required for the decarboxylative condensation [19]. All the ACP domains display the conserved L/IG(x)DS motif with Ser being essential for 4′-phopspho-panthehenylation [20]. The substrate specificity of the AT domains was predicted by sequence multiple alignments and comparison of key residues to published data [21]. AT3–8 and AT15–16 were predicted to recognize malonyl-CoA as substrate, which is the same as that of AT9–14 [18], conversely AT0–2 were predicted to be specific for methylmalonyl-CoA (Fig. 4).

All the modules harbor KR domains with the conserved NADP(H)-binding motif and the catalytic triad KSY [22], among which KR9–11 and KR12–14 were previously predicted to be A-type and B-type, respectively [18]. We analyzed all the other KRs and found that: (i) KR1–3 and KR15–16 belong to B-type with the conserved LDD-motif in KR1–3, or the replaced LED-motif in KR15–16, thus putatively generating *R*-configured alcohols; (ii) KR6–7 possess a Trp residue and are A-type, catalyzing the formation of *S*-configured alcohols; (iii) conversely, no signature motif was seen in KR4–5 and KR8, and thus it is difficult to predict the corresponding products (Additional file 1: Table S4). In addition to modules 12–14, functional DH domains featuring the conserved consensus sequence HxxxGxxxP [23] were found for modules 1–3 and 15–16 which catalyze elimination of water from *R*-configured hydroxyl group to form *E*-configured double bond. Only one ER domain was identified in module 1 with the conserved motif LxHxxxGGVGxxAxxxA [24].

Thus the entire assembly line for RDMs was proposed as shown in Fig. 3. RdmG–J catalyze the RDMs biosynthesis by carrying out 16 rounds of decarboxylative condensation using malonyl-CoA as the starter unit (loading module), two methylmalonyl-CoA (module 1 and 2), and fourteen malonyl-CoA (module 3–16) as extender units. A full-length polyketide intermediate is offloaded and cyclized by the dedicated RdmJ-TE domain, generating RDM A. The present analysis of RdmG–J well supported the proposed pathway but with the following two inconsistencies: (i) phylogenetic analysis supported AT0 to be specific for methylmalonyl-CoA (Fig. 4), however, the skeleton of RDMs indicates AT0 is supposed to utilize malonyl-CoA instead (Fig. 3); (ii) albeit the presence of an active DH domain in module 2, it is most likely “skipped” due to unknown reason (Fig. 3).

### Genes encoding regulators

There are five ORFs (*rdmA*, *rdmC*, *rdmD*, *rdmE* and *rdmF*) located upstream of the PKS genes. As shown in Table 1, RdmA is a putatively XRE family transcriptional regulator with 57% identity/65% similarity to ASD26_RS06115 (WP_056788122.1) from *Streptomyces* sp. Root1319. RdmC–E are putative LuxR family two-component system (TCS) regulators homologous to KasWX from the biosynthetic gene cluster of kasugamycin in *Streptomyces kasugaensis* [25]; RdmC is the homolog of the response regulator KasW, containing a signal receiver domain REC and a helix-turn-helix (HTH) DNA binding domain; interestingly, RdmD and RdmE display homology to different regions of the same protein, the sensor kinase KasX (BAF79687.1). RdmF is located right adjacent to the PKSs and harbors a PAS fold motif (pfam08448) at N-terminus and a LuxR-type HTH DNA binding domain at C-terminus, with 83% identity/89% similarity to a hypothetical protein AOM46_RS26735 (WP_062215992.1) from *Streptomyces* sp. NBRC 109706, and 38% identity/51% similarity to NysRIV (AAF71781.1) from the nystatin gene cluster. In comparison with the regulators in the other polyene biosynthetic gene clusters, PAS-LuxR superfamily regulators are well-conserved and function in positive manner [26]; conversely, no homologs of RdmA and RdmC–E were found in these clusters.

### Functional confirmation and determination of cluster boundaries of the *rdm* gene cluster

**Fig. 5:**
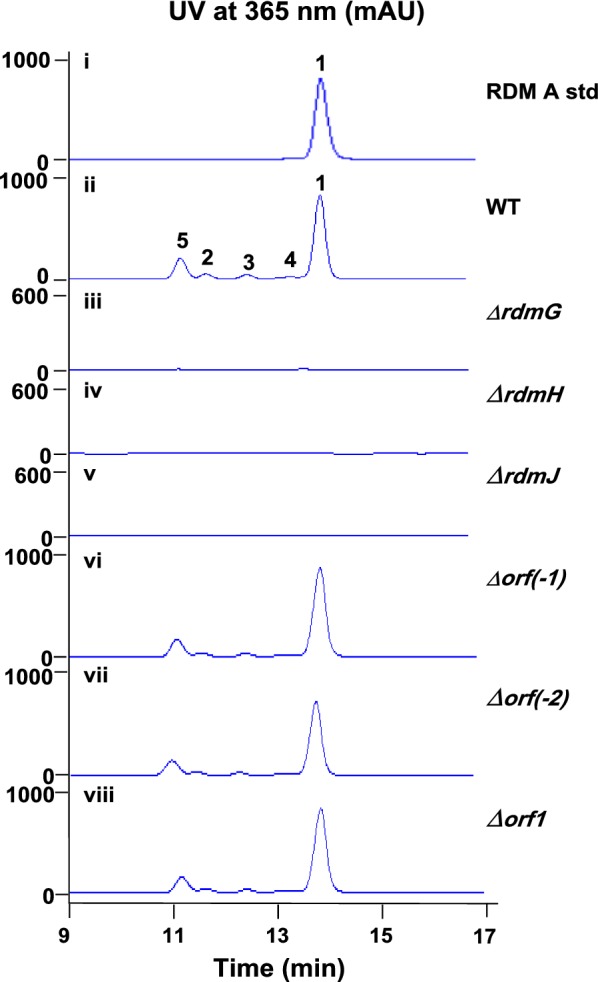
HPLC analysis of the fermentation products from (i) the standard of RDM A; (ii) the wild type strain (WT); (iii) Δ*rdmG*; (iv) Δ*rdmH*; (v) Δ*rdmJ*; (vi) *orf(*−*1)*; (vii) *orf(*−*2)*; (viii) *orf1*

To detect if this locus is indeed involved in RDM biosynthesis, gene inactivation was performed. The PKS genes *rdmG*, *rdmH* and *rdmJ* were replaced with the apramycin resistance cassette, respectively (Additional file 1: Figures S1–S3). Fermentations of the resulting mutants were carried out followed by HPLC and LC–MS analysis (Additional file 1: Figure S4). The results showed that *∆rdmG*, *∆rdmH* and *∆rdmJ* all abolished production of RDMs as expected (Fig. 5iii–v), confirming this locus is indeed involved in RDMs biosynthesis. Based on the sequence analysis result, *orf(*−*1)* and *orf(*−*2)*, which are located upstream of the regulatory gene *rdmA* and putatively encode a methyltransferase and a FAD-binding monooxygenase (Table 1), respectively, were inactivated to determine the upstream boundary (Additional file 1: Figures S5, S6). Orf1 is homologous to the hypothetical protein AMJ94_18775 (KPK85730.1) from *Deltaproteobacteria bacterium* SM23_61 and is interestingly split into two proteins AOK24_RS06255 and AOK24_RS06250 in *S. niveiscabiei* (Table 1). Orf1 was inactivated to determine the downstream boundary (Additional file 1: Figure S7). HPLC analysis results showed that no impact was observed for all the three mutants (Fig. 5vi–viii), suggesting they are probably located outside of the gene cluster.

### A complex regulatory system featuring a negative regulator RdmA and a positive regulator RdmF

**Fig. 6:**
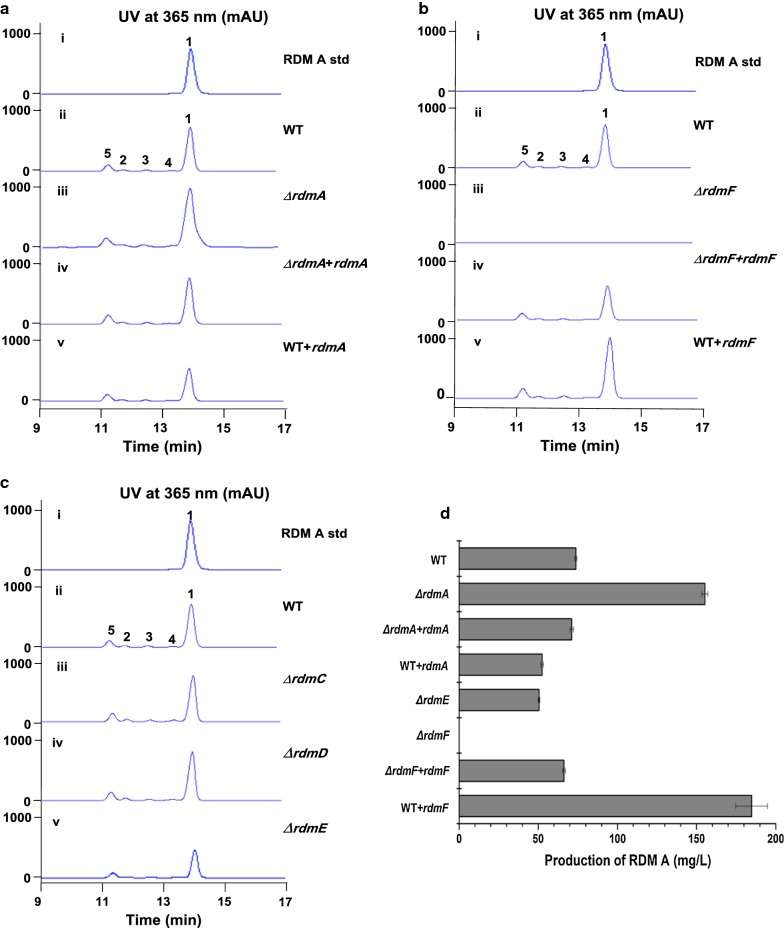
In vivo function of the regulatory genes *rdmACDEF*. **a** HPLC analysis of the fermentation products from (i) the standard of RDM A; (ii) WT; (iii) Δ*rdmA*; (iv) Δ*rdmA* complemented (Δ*rdmA *+ *rdmA*); and (v) *rdmA* overexpression (WT + *rdmA*) strains. **b** HPLC analysis of the fermentation products from (i) the standard of RDM A; (ii) WT; (iii) Δ*rdmF*; (iv) Δ*rdmF *+ *rdmF*; and (v) WT + *rdmF*. **c** HPLC analysis of the fermentation products from gene inactivation mutant strains of *rdmCDE.* (i) the standard of RDM A; (ii) WT; (iii) Δ*rdmC*; (iv) Δ*rdmD*; (v) Δ*rdmE.* **d** Production of RDM A (**1**) in the *rdmAEF*-engineered strains

To probe the function of the regulatory genes, *rdmA* and *rdmC*–*F* were each inactivated as described in the “Methods” section. Fermentations and HPLC analysis were carried out for the wild type strain and the confirmed mutants. The production of RDM A was quantified in each strain as described in “Methods” section, and the results showed: (i) inactivation of *rdmA* led to increased production of RDM A by ~ 2.0-fold, reaching a considerable yield of 155.3 ± 1.89 mg/L, as compared to 73.8 ± 0.76 mg/L in the wild type strain (Fig. 6aiii, d), suggesting RdmA functions as a negative regulator; (ii) in contrast, inactivation of *rdmF* completely abolished the production of RDMs (Fig. 6biii, d), supporting it to be a positive regulator; (iii) while no obvious influences were observed for the *∆rdmC* (Fig. 6ciii) and *∆rdmD* (Fig. 6civ) strains, inactivation of *rdmE* resulted in moderately decreased production of RDM A to 50.5 ± 0.49 mg/L (Fig. 6cv, d).

To further confirm the function of *rdmA* and *rdmF*, genetic complementation and gene overexpression were performed. HPLC analysis results indicated that the genetically complemented strains of *∆rdmA* (Fig. 6aiv, d) and *∆rdmF* almost restore the production of RDMs (Fig. 6biv, d) to levels comparable to those in the wild type strain (Fig. 6a, bii). Overexpression of *rdmA* decreased the production of RDM A by ~ 1.4-fold to a yield of 52.5 ± 0.82 mg/L (Fig. 6av, d), and conversely, overexpression of *rdmF* enhanced the titer of RDM A remarkably by ~ 2.5-fold, reaching 184.8 ± 9.93 mg/L (Fig. 6bv, d), supporting their contrary action of manners.

### Other genes in the *rdm* cluster involved in RDM biosynthesis

**Fig. 7:**
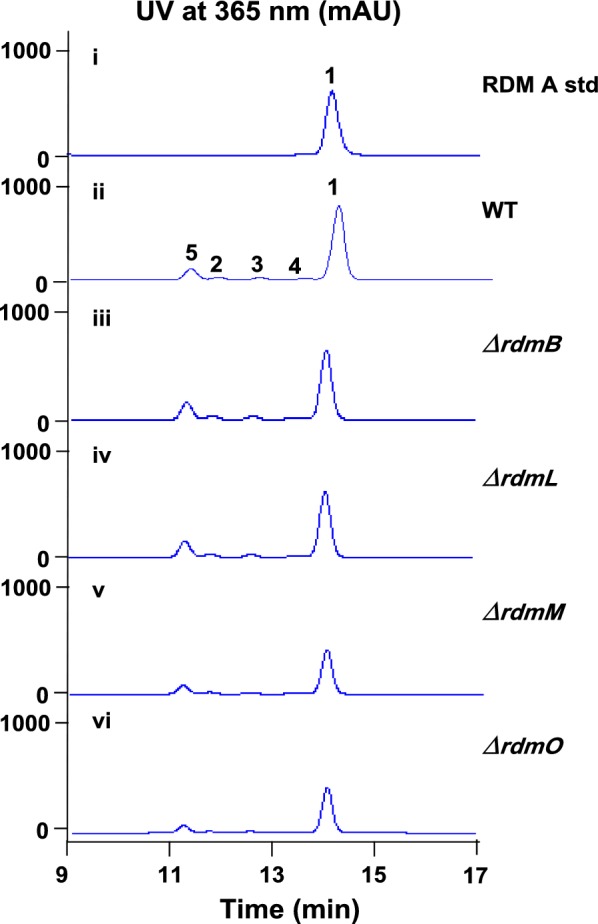
HPLC analysis of the fermentation products from gene inactivation mutant strains of *rdmBLMO.* (i) the standard of RDM A; (ii) WT; (iii) Δ*rdmB*; (iv) Δ*rdmL*; (v) Δ*rdmM*; (vi) Δ*rdmO*

As indicated in Table 1, in addition to the five regulatory genes, a putative methyltransferase gene *rdmB* is found upstream of the PKS genes. To detect if *rdmB* is related to RDM biosynthesis, *rdmB* was inactivated, resulting in no influence at all on the production of RDMs (Fig. 7iii), indicating it might be unnecessary for RDM biosynthesis. The downstream genes adjacent to the PKS genes exhibit considerably high homology to genes from *Streptomyces niveiscabiei*, with RdmK-Orf3 being homologous to AOK24_RS06280-AOK24_RS06240 (Table 1), respectively. We further searched the neighboring region of this locus in the genome of *S. niveiscabiei* (NZ_LIRL00000000.1), and found the presence of a hypothetical protein AOK24_RS06285 (WP_055719057.1) containing a conserved family A glycosyltransferase motif instead of PKSs. *rdmK* encodes a putative MFS transporter, suggesting it is probably a resistance gene; RdmL harbors a conserved ferritin-like motif, which participate in a range of functions such as iron regulation, mono-oxygenation, and reactive radical production; interestingly, *rdmM* encodes a PaaI family TE belonging to the TE13 family, instead of a type II TE belonging to the TE18 family normally found in PKS gene clusters [27]; the putative acyl-CoA synthetase RdmO is possibly involved in substrate formation for the PKSs. To detect if these genes are involved in RDMs biosynthesis, *rdmL*, *rdmM* and *rdmO* were inactivated, respectively. As shown in Fig. 7, no impact was observed when *rdmL* was inactivated (iv), but the production of RDMs in *∆rdmM* (v) and *∆rdmO* (vi) was obviously decreased by ~ 1.4-fold as compared with that in the wild type strain (ii), indicating they are probably involved in the biosynthesis of RDMs. The decreased instead of abolished production of RDMs might be due to presence of homologous genes of *rdmM* and *rdmO* in other places of the genome.

## Discussion

High throughput sequencing has greatly promoted genome-directed natural product discovery as well as understanding of underlying biosynthetic machinery. Herein, we identified and characterized the entire biosynthetic gene cluster of the polyene macrolide compound RDMs, and proposed its biosynthetic pathway based on the genetic organization of the *rdm* gene cluster and in vivo gene manipulation, setting the stage for generating novel RDM analogs with improved bioactivities.

The module and domain organization of RdmG–J is co-linear with the RDM structure excluding the predicted superfluous DH domain in module 2 (Fig. 3). In general, KSq-type loading ATs exhibit a strict substrate specificity with selection of either malonyl-CoA or methylmalony-CoA, which is decarboxylated in situ to provide acetyl or propionate starter units for the polyketide initiation [28]. Interestingly, the KSq-type loading AT0 of RdmG is presumably to recognize malonyl-CoA based on the structure of RDM A, however, it is clustered with ATs recognizing methylmalonyl-CoA instead (Fig. 4). Further biochemical experiments would be performed to elucidate the substrate selectivity of RdmAT0.

Stereochemistry determination is always very challenging for the polyol system of polyene macrolides antibiotics. During the PKS assemble line, the hydroxyl groups are formed by KR domains in a stereospecific manner, thereby bioinformatics analysis would contribute to configuration assignment. In the case of RDM A, *S*-configured hydroxyl groups at C-13, C-15, C-17, C-21 and C-23 of RDMs would be expected, resulting respectively from A-type KRs 11, 10, 9, 7 and 6; conversely configurations of hydroxyl groups at C-19, C-25 and C-27 are not predictable being lack of conserved residues. Nevertheless, X-ray crystallography would be necessary to determine their stereochemistry. Being the dominant product, RDM A (**1**) harbors six *trans* (*E*)-double bonds (*Δ*^2^–*Δ*^10^ and *Δ*^28^) (Fig. 1). This is supported by presence of DH domains in module 3, and 12–16, which catalyze *syn* elimination operating on *R*-configured β-hydroxy intermediates (B-type ketoreduction). The occurrence of *cis*-double bonds in RDM B–E (**2**–**5**) might be due to spontaneous *trans*–*cis* isomerization [11].

In comparison to other polyene macrolide gene clusters, such as pimaricin [7], amphotericin [6], candicidin [4, 5], nystatin [8], NPP [10] and tetramycin [9] biosynthetic gene clusters, the *rdm* gene cluster is devoid of mycosamine biosynthetic genes and cytochrome P450 genes, which are involved in tailoring steps after macrocyclization. Many studies have demonstrated that mycosaminyltransferases tolerate structural changes in their aglycones and show moderately strict specificity for NDP-sugar donors [11, 29]. For example, mycosaminyltransferases AmphDI (in the amphotericin gene cluster) and NysDI (in the nystatin gene cluster) are capable to mycosaminylate candicidin and pimaricin aglycones in vivo albeit with less efficiency compared to the cognate enzymes [29]. The broad substrate promiscuity displayed by mycosaminyltransferases could enable addition of sugar moiety onto RDM A, and thus optimize its pharmacological properties, as glycosylation can usually improve water solubility and reduce toxicity [30, 31]. In general, hydroxylation or epoxidation further happens after glycosylation, which is accomplished by cytochrome P450 monooxygenases. These enzymes were demonstrated to be able to recognize substrates with altered structures [32], allowing for possibility of combinatorial engineering of them with the *rdm* biosynthetic gene cluster to generate RDM analogues.

The *rdm* gene cluster seems to have a regulation system different from those of the other reported polyene gene clusters. In addition to the well conserved PAS/LuxR family regulatory gene *rdmF*, the *rdm* cluster also harbors an XRE family transcriptional regulator RdmA and a set of LuxR family two-component system RdmC–E. Interestingly, the sensor kinase gene was split into two genes *rdmD* and *rdmE*, which are homologous to different regions of *kasX* from the kasugamycin gene cluster. Inactivation of *rdmD* had no obvious impact on RDM production, while inactivation of *rdmE* led to decreased production of RDM A by ~ 1.5-fold (Fig. 6c, d), different from KasX acting in a negative manner during kasugamycin biosynthesis [33]. The hierarchical relationships between the *rdmACDEF* genes are under investigation in our lab. Elucidation of the regulation mechanism would contribute to boosting the production of bioactive RDM compounds in a rational way.

## Conclusions

The biosynthetic gene cluster of non-glycosylated polyene antibiotic RDMs was cloned and characterized in vivo. The proposed model for RDM A assembly line agrees well with its chemical structure, and is supported by gene inactivation. A complex regulatory system consisting of 5 genes was characterized by gene inactivation, genetic complementation as well as gene overexpression, leading to yield improvement of RDM A by ~ 2.0- to 2.5-fold. These findings set the stage for further generation of new biologically active RDM derivatives with improved properties and for yield enhancement by genetic engineering.

## Methods

### Bacterial strains, plasmids, and culture conditions

Bacterial strains and plasmids used in this study are listed in Additional file 1: Table S1 and the PCR primers in Additional file 1: Tables S2, S3. *Escherichia coli* strains including DH5α, BW25113/pIJ790 and ET12567/pUZ8002 were cultivated at 37 °C in Luria–Bertani (LB) liquid medium or on LB agar. *S*. *youssoufiensis* OUC6819 and its derivatives were cultured at 30 °C in TSBY (yeast extract 5 g/L, tryptic soy broth 30 g/L, sucrose 103 g/L) for DNA extraction and on International Streptomyces Project Synthetic Salts-Starch Medium (ISP4) agar with 0.5% glycine for genetic manipulation and on R2YE agar for sporulation. For fermentation, spores of the strain *S. youssoufiensis* OUC6819 and its derivatives were inoculated in fermentation medium (soluble starch 10 g/L, glucose 20 g/L, corn syrup 4 g/L, yeast extract 10 g/L, beef extract 3 g/L, MgSO_4_·7H_2_O 0.5 g/L, KH_2_PO_4_ 0.5 g/L, CaCO_3_ 2 g/L, sea salt 30 g/L, pH = 7.0) and incubated at 30 °C, 220 rpm for 7 days in a 250 mL flask. When necessary, the medium was supplemented with apramycin 50 μg/mL, chloramphenicol 25 μg/mL, kanamycin 100 μg/mL, thiostrepton 25 μg/mL, or ampicillin 100 μg/mL. Common biochemicals and chemicals were purchased from commercial sources.

### Genomic library construction and screening

Genomic DNA of *S*. *youssoufiensis* OUC6819 was partially digested with *Sau*3AI, and fragments with the size of 40–50 kb were recovered and dephosphorylated with FastAP (Thermo Scientific, Pittsburgh, USA), and then ligated into SuperCos1 that was pretreated with *Xba*I, dephosphorylated, and digested with *Bam*HI. The ligation product was packaged into lambda particles with the MaxPlax Lambda Packaging Extract (Epicenter, Madison, WI, USA) as per the manufacture’s instruction and plated on *E. coli* Top10. The titer of the primary library was about 5 × 10^6^ cfu per μg of DNA. The primer pairs used for cosmid library screening are listed in Additional file 1: Table S2.

### DNA manipulation and bioinformatic analysis

All DNA manipulations were performed according to standard procedures [34] or manufacturer’s instruction. Plasmid extractions and DNA purification were carried out using commercial kits (OMEGA, BIO-TEK). Both primer synthesis and DNA sequencing were performed at Tsingke Biotech Co. Ltd. (Qingdao, China). ORF assignments and their proposed function were accomplished by using the FramePlot4.0beta (http://nocardia.nih.go.jp/fp4) [35]. Sequence comparisons and database searches were carried out by BLAST algorithm [36] (http://blast.ncbi.nlm.nih.gov/Blast.cgi). Domain analysis were accomplished at SBSPKS [37]. Additional sequence alignments were conducted by ClustalX [38].

### Construction of gene inactivation mutants

Gene inactivation in *S*. *youssoufiensis* OUC6819 was performed using the REDIRECT Technology according to the literature protocol [39]. For the construction of gene mutants, the *aac(3)IV*-*oriT* resistance cassette from pIJ773 or pMT3 was amplified to replace an internal region of the target gene. The target gene disruption plasmids were transformed into *E. coli* ET12567/pUZ8002, and the conjugation between *E. coli* ET12567/pUZ8002 and *S*. *youssoufiensis* OUC6819 was performed using ultrasonic fragmented mycelia as acceptors. The ultrasonic program is set to cycle 20 times with 3 s ON and 4 s OFF. Desired mutants were selected by apramycin-resistant and kanamycin-sensitive phenotype, followed by PCR confirmation (Additional file 1: Table S3).

### Genetic complementation and overexpression

For genetic complementation and overexpression, *rdmA* and *rdmF* was each put under the control of the constitutive promoter P_*gapdh*_ from *S. youssoufiensis* OUC6819. Construction of *rdmA* expression plasmid was performed as follows: P_*gapdh*_ was amplified using primer pair of pGFP/3′-OH phosphorylated pGRP (Additional file 1: Table S3) and was digested with *Eco*RI; the *rdmA* fragment was amplified with the primer pair of rdmAEF/rdmAER (Additional file 1: Table S3) and digested with *Xba*I; and then these two digested fragments were ligated and cloned into the *Eco*RI and *Xba*I sites of pSET152C to give pWLI515 (Additional file 1: Table S1). Similarly, the fragments of *rdmF* were cloned into the same sites of pSET152C to obtain pWLI516 (Additional file 1: Table S1). After confirmation by sequencing, the resulting plasmids were passed through *E. coli* ET12567/pUZ8002 and introduced into the *rdmA* and *rdmF* mutants for genetic complementation or into the wild type *S. youssoufiensis* OUC6819 for overexpression via conjugation.

### Production and analyses of Reedsmycin A

Spores of *Streptomyces* strains were inoculated into 50 mL medium in a 250 mL flask, and were incubated on a rotatory shaker at 30 °C, 220 rpm for 7 days. The fermentation cultures were harvested by centrifugation and the supernatant was extracted twice with an equal volume of ethyl acetate. The precipitate was extracted by acetone and dried in vacuo then merged to ethyl acetate. After the residue was dried in vacuo, the crude extract was dissolved in 1.5 mL methanol, filtered through a 0.2 μm filter, and subjected to HPLC analysis with injection volume of 25 μL for each sample. The HPLC system consisted of Agilent 1260 Infinity Quaternary pumps and a 1260 Infinity diode-array detector. Analytical HPLC was performed on an Eclipse C18 column (5 μ, 4.6 × 150 mm) developed with a linear gradient from 40 to 65% ACN/H_2_O in 15 min followed by an additional 15 min at 100% ACN at flow rate of 1 mL/min and UV detection at 365 nm. The identity of reedsmycin A (RDM A) was unambiguously confirmed by comparison with authentic standard as well as HR-ESI–MS analysis. HR-ESI–MS was carried out on Thermo LTQ-XL mass spectrometer. RDM A was quantified on the basis of peak area at 365 nm using standard curve obtained with RDM A standard. All experiments were repeated at least three times.

### Nucleotide sequence accession number

The sequence of the *rdm* biosynthetic gene cluster has been deposited in GenBank under the accession number MG947597.

## Additional file

**Additional file 1: Table S1.** Bacteria and plasmids used in this study. **Table S2.** The primer pairs used for cosmid library screening. **Table S3.** The primer pairs used for PCR-targeted mutagenesis. **Table S4.** The conserved motifs in the KR domains. **Figure S1.** Inactivation of *rdmG*. **Figure S2.** Inactivation of *rdmH*. **Figure S3**. Inactivation of *rdmJ*. **Figure S4.** The HRMS spectra of RDMs. **Figure S5.** Inactivation of *orf(*−*2)*. **Figure S6.** Inactivation of *orf(*−*1)*. **Figure S7.** Inactivation of *orf1*. **Figure S8.** Inactivation of *rdmA*. **Figure S9.** Inactivation of *rdmB*. **Figure S10.** Inactivation of *rdmC*. **Figure S11.** Inactivation of *rdmD*. **Figure S12.** Inactivation of *rdmE*. **Figure S13.** Inactivation of *rdmF*. **Figure S14.** Inactivation of *rdmL*. **Figure S15.** Inactivation of *rdmM*. **Figure S16.** Inactivation of *rdmO*.

## Abbreviations

PKS: polyketide synthase
RDM: reedsmycin
ORF: open reading frame
TE: thioesterase
ACP: acyl carrier protein
KS: ketosynthase
AT: acyl transferase
DH: dehydratase
KR: ketoreductase
ER: enoylreductase
TCS: two-component system
HPLC: high-pressure liquid chromatography
UV: ultra violet
LB: Luria–Bertani
TSBY: tryptic soy broth supplement with yeast extract
ISP4: International *Streptomyces* Project Synthetic Salts-Starch Medium

## References

[1] Campoy S, Adrio JL (2017). Antifungals. Biochem Pharmacol.

[2] Zotchev SB (2003). Polyene macrolide antibiotics and their applications in human therapy. Curr Med Chem.

[3] Anderson TM, Clay MC, Cioffi AG, Diaz KA, Hisao GS, Tuttle MD, Nieuwkoop AJ, Comellas G, Maryum N, Wang S (2014). Amphotericin forms an extramembranous and fungicidal sterol sponge. Nat Chem Biol.

[4] Chen S, Huang X, Zhou X, Bai L, He J, Jeong KJ, Lee SY, Deng Z (2003). Organizational and mutational analysis of a complete FR-008/candicidin gene cluster encoding a structurally related polyene complex. Chem Biol.

[5] Campelo AB, Gil JA (2002). The candicidin gene cluster from *Streptomyces griseus* IMRU 3570. Microbiology.

[6] Caffrey P, Lynch S, Flood E, Finnan S, Oliynyk M (2001). Amphotericin biosynthesis in *Streptomyces nodosus*: deductions from analysis of polyketide synthase and late genes. Chem Biol.

[7] Aparicio JF, Colina AJ, Ceballos E (1999). Martín JF. The biosynthetic gene cluster for the 26-membered ring polyene macrolide pimaricin. J Biol Chem.

[8] Fjærvik E, Zotchev SB (2005). Biosynthesis of the polyene macrolide antibiotic nystatin in *Streptomyces noursei*. Appl Microbiol Biotechnol.

[9] Cao B, Yao F, Zheng X, Cui D, Shao Y, Zhu C, Deng Z, You D (2012). Genome mining of the biosynthetic gene cluster of the polyene macrolide antibiotic tetramycin and characterization of a P450 monooxygenase involved in the hydroxylation of the tetramycin B polyol segment. ChemBioChem.

[10] Kim BG, Lee MJ, Seo J, Hwang YB, Lee MY, Han K, Sherman DH, Kim ES (2009). Identification of functionally clustered nystatin-like biosynthetic genes in a rare actinomycetes, *Pseudonocardia autotrophica*. J Ind Microbiol Biotechnol.

[11] Caffrey P, De Poire E, Sheehan J, Sweeney P (2016). Polyene macrolide biosynthesis in streptomycetes and related bacteria: recent advances from genome sequencing and experimental studies. Appl Microbiol Biotechnol.

[12] Power P, Dunne T, Murphy B, Lochlainn LN, Rai D, Borissow C, Rawlings B, Caffrey P (2008). Engineered synthesis of 7-oxo- and 15-deoxy-15-oxo-amphotericins: insights into structure-activity relationships in polyene antibiotics. Chem Biol.

[13] Carmody M, Murphy B, Byrne B, Power P, Rai D, Rawlings B, Caffrey P (2005). Biosynthesis of amphotericin derivatives lacking exocyclic carboxyl groups. J Biol Chem.

[14] Brautaset T, Sletta H, Nedal A, Borgos SEF, Degnes KF, Bakke I, Volokhan O, Sekurova ON, Treshalin ID, Mirchink EP (2008). Improved antifungal polyene macrolides via engineering of the nystatin biosynthetic genes in *Streptomyces noursei*. Chem Biol.

[15] Chen S, Mao X, Shen Y, Zhou Y, Li J, Wang L, Tao X, Yang L, Wang Y, Zhou X (2009). Tailoring the P450 monooxygenase gene for FR-008/candicidin biosynthesis. Appl Environ Microbiol.

[16] Seco EM, Fotso S, Laatsch H, Malpartida F (2005). A tailoring activity is responsible for generating polyene amide derivatives in *Streptomyces diastaticus* var. 108. Chem Biol.

[17] Kim HJ, Kang SH, Choi SS, Kim ES (2017). Redesign of antifungal polyene glycosylation: engineered biosynthesis of disaccharide-modified NPP. Appl Microbiol Biotechnol.

[18] Che Q, Li T, Liu X, Yao T, Li J, Gu Q, Li D, Li W, Zhu T (2015). Genome scanning inspired isolation of reedsmycins A–F, polyene-polyol macrolides from *Streptomyces* sp. CHQ-64. RSC Adv.

[19] Li W, Ju J, Rajski SR, Osada H, Shen B (2008). Characterization of the tautomycin biosynthetic gene cluster from *Streptomyces spiroverticillatus* unveiling new insights into dialkylmaleic anhydride and polyketide biosynthesis. J Biol Chem.

[20] Staunton J, Weissman KJ (2001). Polyketide biosynthesis: a millennium review. Nat Prod Rep.

[21] Yadav G, Gokhale RS, Mohanty D (2003). Computational approach for prediction of domain organization and substrate specificity of modular polyketide synthases. J Mol Biol.

[22] Reid R, Piagentini M, Rodriguez E, Ashley G, Viswanathan N, Carney J, Santi DV, Hutchinson CR, McDaniel R (2003). A model of structure and catalysis for ketoreductase domains in modular polyketide synthases. Biochemistry.

[23] Bevitt DJ, Cortes J, Haydock SF, Leadlay PF (1992). 6-Deoxyerythronolide-B synthase 2 from *Saccharopolyspora erythraea*. FEBS J.

[24] Kakavas SJ, Katz L, Stassi D (1997). Identification and characterization of the niddamycin polyketide synthase genes from *Streptomyces caelestis*. J Bacteriol.

[25] Kasuga K, Sasaki A, Matsuo T, Yamamoto C, Minato Y, Kuwahara N, Fujii C, Kobayashi M, Agematu H, Tamura T (2017). Heterologous production of kasugamycin, an aminoglycoside antibiotic from *Streptomyces kasugaensis*, in *Streptomyces lividans* and *Rhodococcus erythropolis* L-88 by constitutive expression of the biosynthetic gene cluster. Appl Microbiol Biotechnol.

[26] Vicente CM, Payero TD, Santos-Aberturas J, Barreales EG, de Pedro A, Aparicio JF (2015). Pathway-specific regulation revisited: cross-regulation of multiple disparate gene clusters by PAS-LuxR transcriptional regulators. Appl Microbiol Biotechnol.

[27] Cantu DC, Chen Y, Reilly PJ (2010). Thioesterases: a new perspective based on their primary and tertiary structures. Protein Sci.

[28] Long PF, Wilkinson CJ, Bisang CP, Cortés J, Dunster N, Oliynyk M, McCormick E, McArthur H, Mendez C, Salas JA (2002). Engineering specificity of starter unit selection by the erythromycin-producing polyketide synthase. Mol Microbiol.

[29] Murphy B, Anderson K, Borissow C, Caffrey P, Griffith G, Hearn J, Ibrahim O, Khan N, Lamburn N, Lee M, Pugh K, Rawlings B (2010). Isolation and characterisation of amphotericin B analogues and truncated polyketide intermediates produced by genetic engineering of *Streptomyces nodosus*. Org Biomol Chem.

[30] Kren V, Martínková L (2001). Glycosides in medicine:“The role of glycosidic residue in biological activity”. Curr Med Chem.

[31] Gray KC, Palacios DS, Dailey I, Endo MM, Uno BE, Wilcock BC, Burke MD (2012). Amphotericin primarily kills yeast by simply binding ergosterol. Proc Natl Acad Sci USA.

[32] Kong D, Lee MJ, Lin S, Kim ES (2013). Biosynthesis and pathway engineering of antifungal polyene macrolides in actinomycetes. J Ind Microbiol Biotechnol.

[33] Zhu C, Kang Q, Bai L, Cheng L, Deng Z (2016). Identification and engineering of regulation-related genes toward improved kasugamycin production. Appl Microbiol Biotechnol.

[34] Sambrook J, Fritsch EF, Maniatis T (1989). Molecular cloning: a laboratory manual.

[35] Ishikawa J, Hotta K (1999). FramePlot: a new implementation of the frame analysis for predicting protein-coding regions in bacterial DNA with a high G+C content. FEMS Microbiol Lett.

[36] McGinnis S, Madden TL (2004). BLAST: at the core of a powerful and diverse set of sequence analysis tools. Nucleic Acids Res.

[37] Anand S, Prasad M, Yadav G, Kumar N, Shehara J, Ansari MZ, Mohanty D (2010). SBSPKS: structure based sequence analysis of polyketide synthases. Nucleic Acids Res.

[38] Larkin MA, Blackshields G, Brown NP, Chenna R, Mcgettigan PA, Mcwilliam H, Valentin F, Wallace IM, Wilm A, Lopez R (2007). Clustal W and Clustal X version 2.0. Bioinformatics.

[39] Gust B, Chandra G, Jakimowicz D, Yuqing T, Bruton CJ, Chater KF (2004). Lambda red-mediated genetic manipulation of antibiotic-producing *Streptomyces*. Adv Appl Microbiol.

