# Supplementary material for: Characterization of the biosynthetic gene cluster of the polyene macrolide antibiotic reedsmycins from a marine-derived Streptomyces strain
Source: Microb Cell Fact. 2018 Jun 19;17:98. doi: 10.1186/s12934-018-0943-6 (PMC6006980; doi:10.1186/s12934-018-0943-6)
Supplement: Supplementary file 1 — Additional file 1: Table S1. Bacteria and plasmids used in this study. Table S2. The primer pairs used for cosmid library screening. Table S3. The primer pairs used for PCR-targeted mutagenesis. Table S4. The conserved motifs in the KR domains. Figure S1. Inactivation of rdmG. Figure S2. Inactivation of rdmH. Figure S3. Inactivation of rdmJ. Figure S4. The HRMS spectra of RDMs. Figure S5. Inactivation of orf(−2). Figure S6. Inactivation of orf(−1). Figure S7. Inactivation of orf1. Figure S8. Inactivation of rdmA. Figure S9. Inactivation of rdmB. Figure S10. Inactivation of rdmC. Figure S11. Inactivation of rdmD. Figure S12. Inactivation of rdmE. Figure S13. Inactivation of rdmF. Figure S14. Inactivation of rdmL. Figure S15. Inactivation of rdmM. Figure S16. Inactivation of rdmO. [file 12934_2018_943_MOESM1_ESM.docx]

**Characterization of the biosynthetic gene cluster of the polyene macrolide antibiotic reedsmycins from a marine-derived *Streptomyces* strain**

Tingting Yao^1#^, Zengzhi Liu^1#^, Tong Li^1^, Hui Zhang^1^, Jing Liu^1^, Huayue Li^1,2^, Qian Che^1,2^, Tianjiao Zhu^1,2^, Dehai Li^1,2^, Wenli Li^1,2^*

^1^ Key Laboratory of Marine Drugs, Ministry of Education of China, School of Medicine and Pharmacy, Ocean University of China, Qingdao 266003, China

^2^ Laboratory for Marine Drugs and Bioproducts of Qingdao National Laboratory for Marine Science and Technology, Qingdao 266237, China

^#^ Tingting Yao and Zengzhi Liu contributed equally to this work.

***** Author to whom correspondence should be addressed

Email addresses:

TY: yaotingting0920@126.com

ZL: [liuzz1990@outlook.com](mailto:liuzz1990@outlook.com)

TL: [oaixlittle@163.com](mailto:oaixlittle@163.com)

HZ: [xiazixue2008@126.com](mailto:xiazixue2008@126.com)

JL: liujing900908@163.com

HL: lihuayue@ouc.edu.cn

QC: Cheqian1396@sina.com

TZ: [zhutj@ouc.edu.cn](mailto:zhutj@ouc.edu.cn)

DL: dehaili@ouc.edu.cn

WL: [liwenli@ouc.edu.cn](mailto:liwenli@ouc.edu.cn)

**Table of contents**

| No. |  | Page |
| --- | --- | --- |
| 1． | **Table S1.** Bacteria and plasmids used in this study. | S3 |
| 2. | **Table S2.** The primer pairs used for cosmid library screening. | S4 |
| 3. | **Table S3.** The primer pairs used for PCR-targeted mutagenesis. | S5 |
| 4. | **Table S4.** The conserved motifs in the KR domains. | S6 |
| 5. | **Figure S1**. Inactivation of *rdmG*. | S7 |
| 6. | **Figure S2.** Inactivation of *rdmH*. | S7 |
| 7. | **Figure S3.** Inactivation of *rdmJ*. | S8 |
| 8 | **Figure S4.** The HRMS spectra of RDMs. | S8 |
| 8. | **Figure S5.** Inactivation of *orf(-2)*. | S11 |
| 10. | **Figure S6.** Inactivation of *orf(-1)*. | S11 |
| 11. | **Figure S7.** Inactivation of *orf1*. | S12 |
| 12. | **Figure S8.** Inactivation of *rdmA*. | S12 |
| 13. | **Figure S9.** Inactivation of *rdmB*. | S13 |
| 14. | **Figure S10.** Inactivation of *rdmC*. | S13 |
| 15. | **Figure S11.** Inactivation of *rdmD*. | S14 |
| 16. | **Figure S12.** Inactivation of *rdmE*. | S14 |
| 17.  18  19  20  21 | **Figure S13.** Inactivation of *rdmF*.  **Figure S14.** Inactivation of *rdmL*.  **Figure S15.** Inactivation of *rdmM*.  **Figure S16.** Inactivation of *rdmO*.  **References** | S15  S15  S16  S16  S17 |

**Table S1.** Bacteria and plasmids used in this study.

| Strains or plasmids | Description | Reference or source |
| --- | --- | --- |
| **Strains** |  |  |
| *E. coli* Top10 | Host strain of cosmid vector SuperCos1 | Invitrogen |
| *E. coli* DH5*a* | Host strain for general cloning | Stratagene |
| *E. coli* ET12567/pUZ8002 | Host strain for conjugation | [1] |
| *E. coli* BW25113/pIJ790 | Host strain for PCR-targeting | [2] |
| WT | *Streptomyces youssoufiensis* OUC6819, wild type harboring the *rdm* locus, RDM producer | [3] |
| *Δorf(-2)* | *Orf(-2)* inactivation mutant of *S. youssoufiensis* OUC6819 | This study |
| *Δorf(-1)* | *Orf(-1)* inactivation mutant of *S. youssoufiensis* OUC6819 | This study |
| *ΔrdmA* | *rdmA* inactivation mutant of *S. youssoufiensis* OUC6819 | This study |
| *ΔrdmB* | *rdmB* inactivation mutant of *S. youssoufiensis* OUC6819 | This study |
| *ΔrdmC* | *rdmC* inactivation mutant of *S. youssoufiensis* OUC6819 | This study |
| *ΔrdmD* | *rdmD* inactivation mutant of *S. youssoufiensis* OUC6819 | This study |
| *ΔrdmE* | *rdmE* inactivation mutant of *S. youssoufiensis* OUC6819 | This study |
| *ΔrdmF* | *rdmF* inactivation mutant of *S. youssoufiensis* OUC6819 | This study |
| *ΔrdmG* | *rdmG* inactivation mutant of *S. youssoufiensis* OUC6819 | This study |
| *ΔrdmH* | *rdmH* inactivation mutant of *S. youssoufiensis* OUC6819 | This study |
| *ΔrdmJ* | *rdmJ* inactivation mutant of *S. youssoufiensis* OUC6819 | This study |
| *ΔrdmL* | *rdmL* inactivation mutant of *S. youssoufiensis* OUC6819 | This study |
| *ΔrdmM* | *rdmM* inactivation mutant of *S.youssoufiensis* OUC6819 | This study |
| *ΔrdmO* | *rdmO* inactivation mutant of *S. youssoufiensis* OUC6819 | This study |
| *Δorf1* | *orf1* inactivation mutant of *S. youssoufiensis* OUC6819 | This study |
| **Plasmids** |  |  |
| SuperCosI | Amp^R^, Kan^R^ , cosmid vector | Stratagene |
| pIJ773 | Apr^R^, source of *acc(3)IV*-*oriT* cassette | [4] |
| pMT3 | Apr^R^, source of *acc(3)IV*-*tsr*-*oriT* cassette | [5] |
| pIJ790 | Cm^R^, λ RED recombination plasmid | [4] |
| pSET152C | pSET152 derivative, with insertion of the *neo* gene from SuperCos1 at the sites of *Apa*I and *Sgr*AI | Our laboratory |
| pWLI511 | cosmid harboring partial *rdm* biosynthetic genes from *S. youssoufiensis* OUC6819 | This study |
| pWLI512 | cosmid harboring partial *rdm* biosynthetic genes from *S. youssoufiensis* OUC6819 | This study |
| pWLI513 | cosmid harboring partial *rdm* biosynthetic genes from *S. youssoufiensis* OUC6819 | This study |
| pWLI514 | cosmid harboring partial *rdm* biosynthetic genes from *S. youssoufiensis* OUC6819 | This study |
| pWLI515 | pSET152C derivative harboring *rdmA* under the control of P_gapdh_ | This study |
| pWLI516 | pSET152C derivative harboring *rdmF* under the control of P_gapdh_ | This study |
| pWLI517 | pWLI511 derivative where *orf(-2)* was replaced with *acc(3)IV*-*oriT* cassette | This study |
| pWLI518 | pWLI511 derivative where *orf(-1)* was replaced with *acc(3)IV*-*oriT* cassette | This study |
| pWLI519 | pWLI512 derivative where *rdmA* was replaced with *acc(3)IV*-*oriT* cassette | This study |
| pWLI520 | pWLI512 derivative where *rdmB* was replaced with *acc(3)IV*-*oriT* cassette | This study |
| pWLI521 | pWLI512 derivative where *rdmC* was replaced with *acc(3)IV*-*oriT* cassette | This study |
| pWLI522 | pWLI512 derivative where *rdmD* was replaced with *acc(3)IV*-*oriT* cassette | This study |
| pWLI523 | pWLI512 derivative where *rdmE* was replaced with *acc(3)IV*-*oriT* cassette | This study |
| pWLI524 | pWLI512 derivative where *rdmF* was replaced with *acc(3)IV*-*oriT* cassette | This study |
| pWLI525 | pWLI512 derivative where *rdmG* was replaced with *acc(3)IV*-*oriT* cassette | This study |
| pWLI526 | pWLI512 derivative where *rdmH* was replaced with *acc(3)IV*-*oriT* cassette | This study |
| pWLI527 | pWLI513 derivative where *rdmJ* was replaced with *acc(3)IV*-*oriT* cassette | This study |
| pWLI528 | pWLI513 derivative where *rdmL* was replaced with *acc(3)IV*-*oriT* cassette | This study |
| pWLI529 | pWLI514 derivative where *rdmM* was replaced with *acc(3)IV*-*oriT* cassette | This study |
| pWLI530 | pWLI514 derivative where *rdmO* was replaced with *acc(3)IV*-*oriT* cassette | This study |
| pWLI531 | pWLI514 derivative where *orf1* was replaced with *acc(3)IV*-*oriT* cassette | This study |

**Table S2.** The primer pairs used for cosmid library screening.

| Name | Sequence (5'-3') | |
| --- | --- | --- |
| LS1-FP | | GGCCGACGGTGTGGCGGC |
| LS1-RP | | CTGGCTCGCTCCGCGGTC |
| LS2-FP | | ATGAGGTTTCCCTTCGGAG |
| LS2-RP | | TTGTGGATGCAGCAGGTA |

**Table S3.** The primer pairs used for PCR-targeted mutagenesis^a^.

| gene | Primer pairs used for inactivation (5'-3') |
| --- | --- |
| *orf(-2)* | orf-2MF: GCCGGGCCGACCGGGCCAGAGCCCATGGAGGTACACCATGattccggggatccgtcgacc  orf-2MR: GGCGGGCGACGGAGCAGCGCGGGCGTGCGAGCGGGCTTCAtgtaggctggagctgcttc  orf-2CF: CCAACGACCTCGCCGTACTGCTACT  orf-2CR: GCGACGGCCGTGAACATGTA |
| *orf(-1)* | orf-1MF: ACCACGGATCACGGAAGACATCGGAAGGCAGACGGACATGattccggggatccgtcgacc  orf-1MR: GGCCTCGCCGAGCGCCGTCACCCCCCGCCCTCGTGCCCTAtgtaggctggagctgcttc  orf-1CF: GGGCTTGAGGGCGACCGT  orf-1CR: ACATCGGAAGGCAGACGG |
| *rdmA* | rdmAMF: ACGTGAGCAGGCCACGGTCGGGGCCCACGGTGGAGCACCGGggttcatgtgcagctccat  rdmAMR: ATGTCATCGCTGTGCAGCCTCTCCTTCGAGGGTGGGAAGCGGcggatcttttccgctgca  rdmACF: TGGGGGGACTCAGGAGTGAA  rdmACR: TAACAGGCAGTCCCAGACGCGT  rdmAEF: GTGAGCAGGCCACGGTCGGGGC  rdmAER: GCTCTAGACGGGGCGGCGGATGTCATCG |
| *rdmB* | rdmBMF: TCCGTACCGCACTCAGCACGCGTCTGGGACTGCCTGTTAGattccggggatccgtcgacc  rdmBMR: ATGCGAGCGACGGCGGCGTAGCCGGGGATGTGCTCGTGCTGtgtaggctggagctgcttc  rdmBCF: CTGCACAGCGATGACATC  rdmBCR: CGACTGCACATGAGTGAG |
| *rdmC* | rdmBMF: GGATGATCCTGGGTTCGGCGGAGGATATCGACGTCGTCGGCggttcatgtgcagctccat  rdmBMR: GACTCACACGCCCAGGCCCGCATCGCGGGCCAGCAACGCGGCcggatcttttccgctgca  rdmBCF: CGACCGAACAGCACGAGCACAT  rdmBCR: ATGATCGCTTCGAGACAGCCCG |
| *rdmD* | rdmDMF: TCGACGTCGAAGGACTTCTTCTCGATCAGCTCGCCGTCCTattccggggatccgtcgacc  rdmDMR: CGGAATCGGACTGCTGCTCGTGGTCGCCATCGCGGTGGGCGtgtaggctggagctgcttc  rdmDCF: TGATACCCGCCCGTATGA  rdmDCR: ACTCCCTGATCGTGGAGGT |
| P_gapdh_ | pGFP: CGGAATTCCCGTCGCGGAAAGCTGGC  pGRP: GAACCGATCTCCTCGTTGGTG |
| *rdmE* | rdmEMF: GCAGACCGTTCCCGTCGATGACCGGACCACCTTCACCGCCattccggggatccgtcgacc  rdmEMR: CTGGCGGCCGGCGCCGAGCTGGCGTTGAACAGCAGCGGCCTGtgtaggctggagctgcttc  rdmECF: GTCGATCATGGCCTTGTC  rdmECR: GAAGTCTCGCTGATGGTG |
| *rdmF* | rdmFMF: AGTGACACATGAGGTAAGTTTCCGCACTTCCGAGCTGACGattccggggatccgtcgacc  rdmFMR: GGTCACTCGATGAAATCCGGCCGCACTACGGGGGGCCAGGCtgtaggctggagctgcttc  rdmFCF: CGAAGGGATGACAAGGTCTA  rdmFCR: AGTCGAATTCTCGAACATCG  rdmFEF: GTGACACATGAGGTAAGTT  rdmFER: GCTCTAGATCCTTAGTTCTCGACGGCG |
| *rdmG* | rdmGMF: GGTCACCGGTACCACCGAGGATGCGCGCCACATCGTGTTC attccggggatccgtcgacc  rdmGMR: GCTCGGTCGCGTCGCGGTCGCCGTCCTCGGCGGGCTTCTC tgtaggctggagctgcttc  rdmGCF: GCCGTGACCGCCAGCAACTCATG  rdmGCR: CGCGTACTGCTTCAGCGACTCCG |
| *rdmH* | rdmHMF: GGGCGGCCTGTTGGGGTTGGGTGAGCCAGGGGTTGCGGTGattccggggatccgtcgacc  rdmHMR: CCTGCCCCAGCGCGGTCAGCAGGGCCTGCGCCTCCTCGCGtgtaggctggagctgcttc  rdmHCF: GCGTTGAGGTGCCTGCTGATTCG  rdmHCR: TGCCTGGTCAGCGGCTACTACTCC |
| *rdmJ* | rdmJMF: GCCGAGGGCGAGTCCGTGGTTGGTTCCGGCTTGGTTGGTGattccggggatccgtcgacc  rdmJMR: GGCCCTGCTGACCGGCCCGACGTTAGGAGTCCTGCGCCGCtgtaggctggagctgcttc  rdmJCF: GTTGCGGTGCGTGATGGTGGTGT  rdmJCR: AGTAGTCGCTCTGCCGTGGTAGTTGG |
| *rdmL* | rdmLMF: CGACACGCATCGTCTTCGTCGACTGTCTGCCCGGCACCGTattccggggatccgtcgacc  rdmLMR: GAGCGAGCGCAGCTTGCGCTTGATGTCCCGCGACGATTCGAtgtaggctggagctgcttc  rdmLCF: ACTCCGAAGGGAAACCTC  rdmLCR: GATCAGTGGGTCGAAGGA |
| *rdmM* | rdmMMF: CGAGGCCAGCCCCGCACCACGCCCACCCTGATGAGGAGTGattccggggatccgtcgacc  rdmMMR: GATCTCAAGGACCTTCTCGTGGACGTCCATGCTGGTTCATTtgtaggctggagctgcttc  rdmMCF: GGGACATCAAGCGCAAGCTG  rdmMCR: AAGGCTTTCTCCGTCTTCAA |
| *rdmO* | rdmOMF: AGCTGGTCGCCGAGCTGTGCCGCCAGCGAGCCGCCGCGTGattccggggatccgtcgacc  rdmOMR: AGCGCGTCGGTCATGATGTCTTCCTCCGGTCTGTTGGCCCGtgtaggctggagctgcttc  rdmOCF: CCAGACCGTCGGCGAGTTTC  rdmOCR: GCAGGAGTTCCTCGCGTTGC |
| *orf1* | orf4MF: GCGAGTGACGCGGGCCAACAGACCGGAGGAAGACATCATGattccggggatccgtcgacc  orf4MR: TGAGCGAGTCTGGCCGACTCGCGGTCGACGAAAGCCCTCAtgtaggctggagctgcttc  orf4CF: GAGACCAAGGAGTCTGATC  orf4CR: CCAACATCTACCGCAAGT |

^a^Underlined letters represent nucleotides homologous to the DNA regions internal to target genes. The restriction site of *Xba*I was colored in blue. The primer pairs of MF/MR were used for gene deletion. The primer pairs of CF/CR were used for PCR confirmation of the mutants. The primer pairs of rdmAEF/ER and rdmFEF/ER were used for genetic complementation of *∆rdmA* and *∆rdmF*. pGFP/RP was used for amplification of promoter P*_gapdh_*_._

**Table S4.** The conserved motifs in the KR domains.

| **Domain** | **Motif I** | **Motif II** | **Type** | **Predicted configuration** |
| --- | --- | --- | --- | --- |
| KR1 | TAGV**LDD**GVLSAQ | VMATLGSAGQSN**Y**TAANAF | B | - |
| KR2 | TAGV**LDD**AIMTAL | VAGHLAGFGSGS**Y**SAANAF | B | *R*-OH |
| KR3 | TAGV**LDD**AIMTAL | VAGHLAGFGSGS**Y**SAANAF | B | *E* |
| KR4 | AAGVDVSGLIDDL | VAGVLGGGGVGA**Y**GAANAY | - | *-* |
| KR5 | TAGVDVSGLVDEL | VAGLFGGGGAGA**Y**GAANAY | - | *-* |
| KR6 | AAGVSQNREIQTI | IAAA**W**GSASNGA**Y**AAANAF | A | *S*-OH |
| KR7 | AAGVSHYRLLGDL | IAGV**W**GSAGDGA**Y**AAANAF | A | *S-*OH |
| KR8 | AAGVDVSGLLGEL | VAGVLGGGGAGA**Y**GAANAY | - | *-* |
| KR15 | AAGV**LED**GVFSSL | AAGVLGNAGQAA**Y**GAANAY | B | *E* |
| KR16 | AAGV**LED**GVFSSL | AAGVLGNAGQAA**Y**GAANAY | B | *E* |

**(B)**

**(A)**

**Figure S1.** Inactivation of *rdmG*. (A) Construction of *rdmG* gene inactivation mutant. (B) PCR confirmation of the double-crossover mutant. M: DNA marker; W: *S. youssoufiensis OUC6819* wild type strain; Mutation: *rdmG* gene inactivation mutant.

**(B)**

**(A)**

**Figure S2.** Inactivation of *rdmH*. (A) Construction of *rdmH* gene inactivation mutant. (B) PCR confirmation of the double-crossover mutant. M: DNA marker; W: *S. youssoufiensis* OUC6819 wild type strain; Mutation: *rdmH* gene inactivation mutant.

**(B)**

**(A)**

**Figure S3.** Inactivation of *rdmJ*. (A) Construction of *rdmJ* gene inactivation mutant. (B) PCR confirmation of the double-crossover mutant. M: DNA marker; W: *S. youssoufiensis* OUC6819 wild type strain; Mutation: *rdmJ* gene inactivation mutant.

**Figure S4.** The HRMS spectra of RDMs.


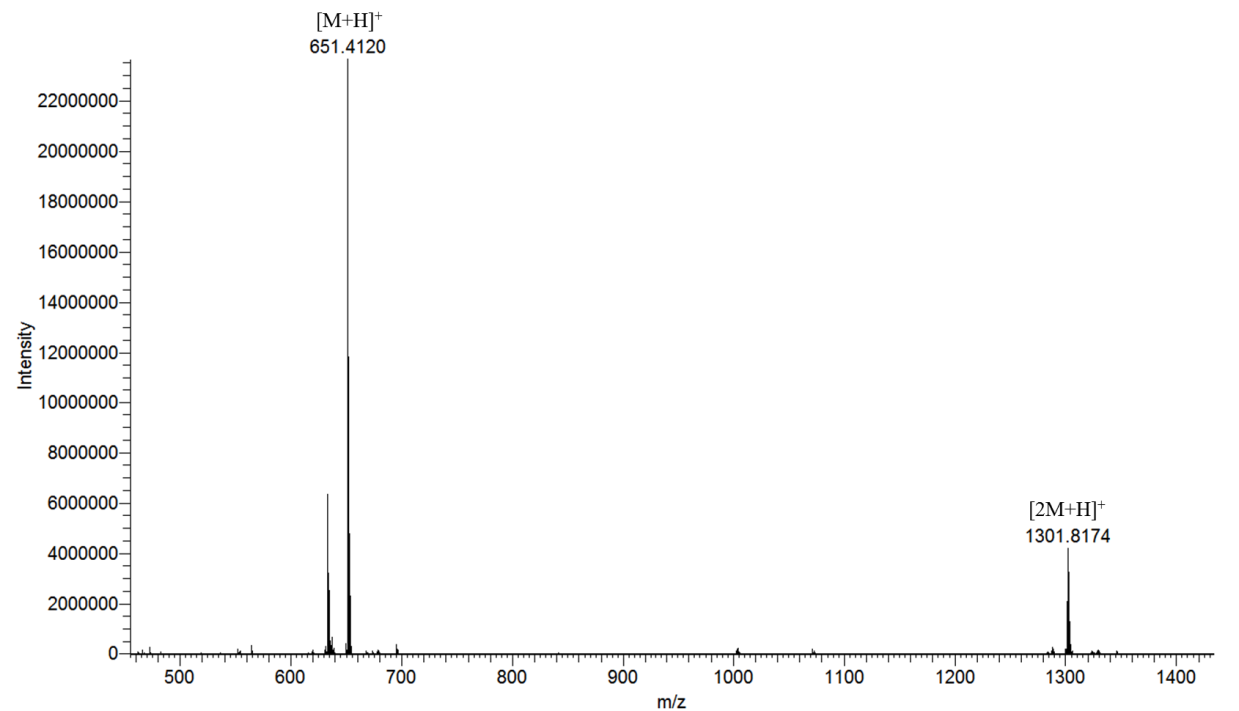


**Figure S4A.** The HRMS spectrum of **5**.


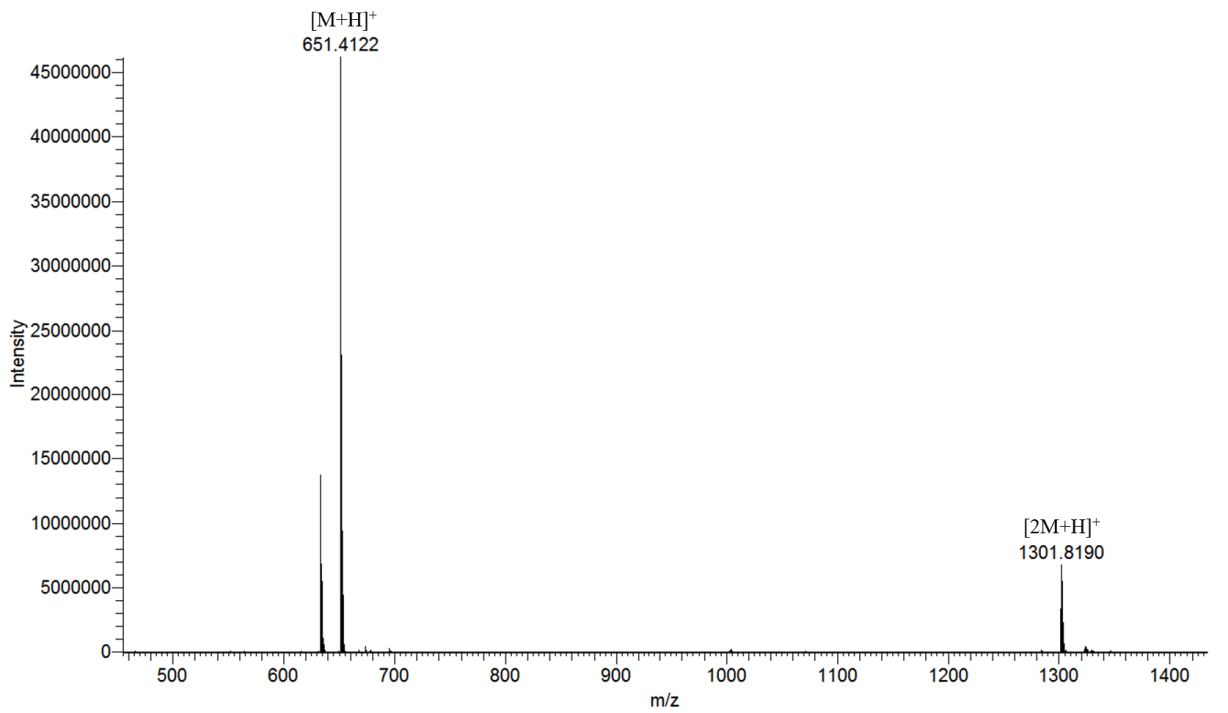


**Figure S4B.** The HRMS spectrum of **2**.


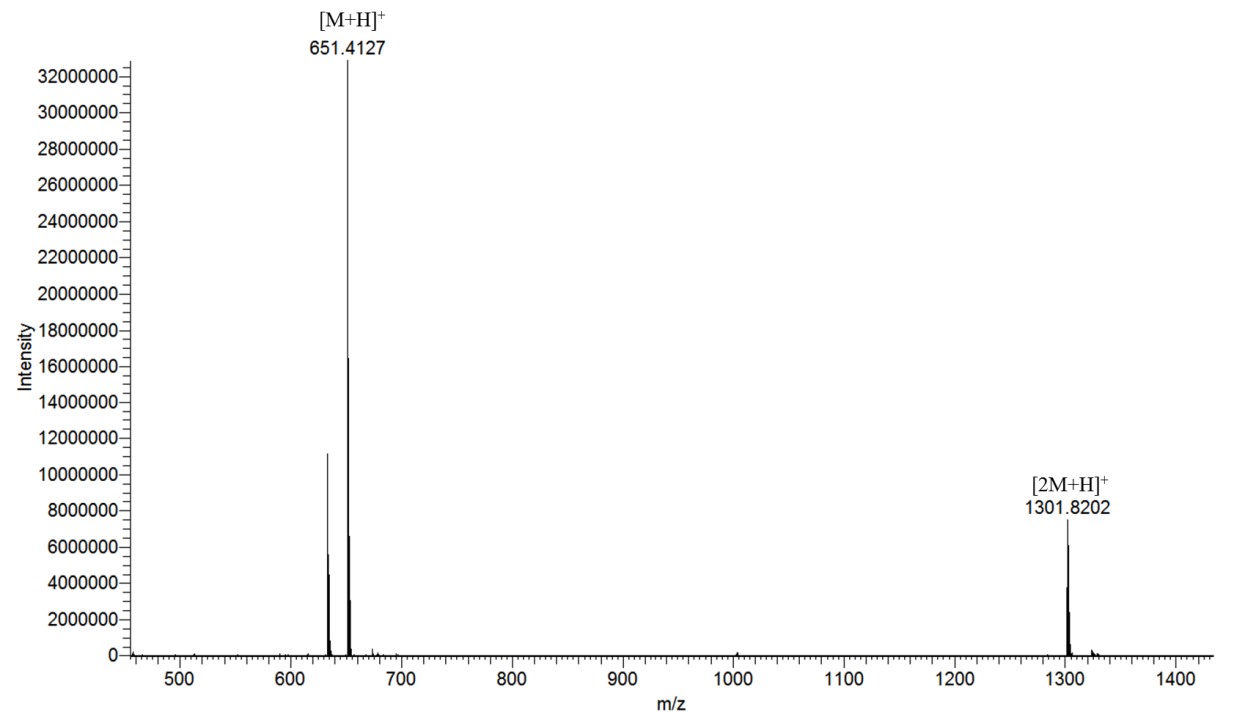


**Figure S4C.** The HRMS spectra of **3**.


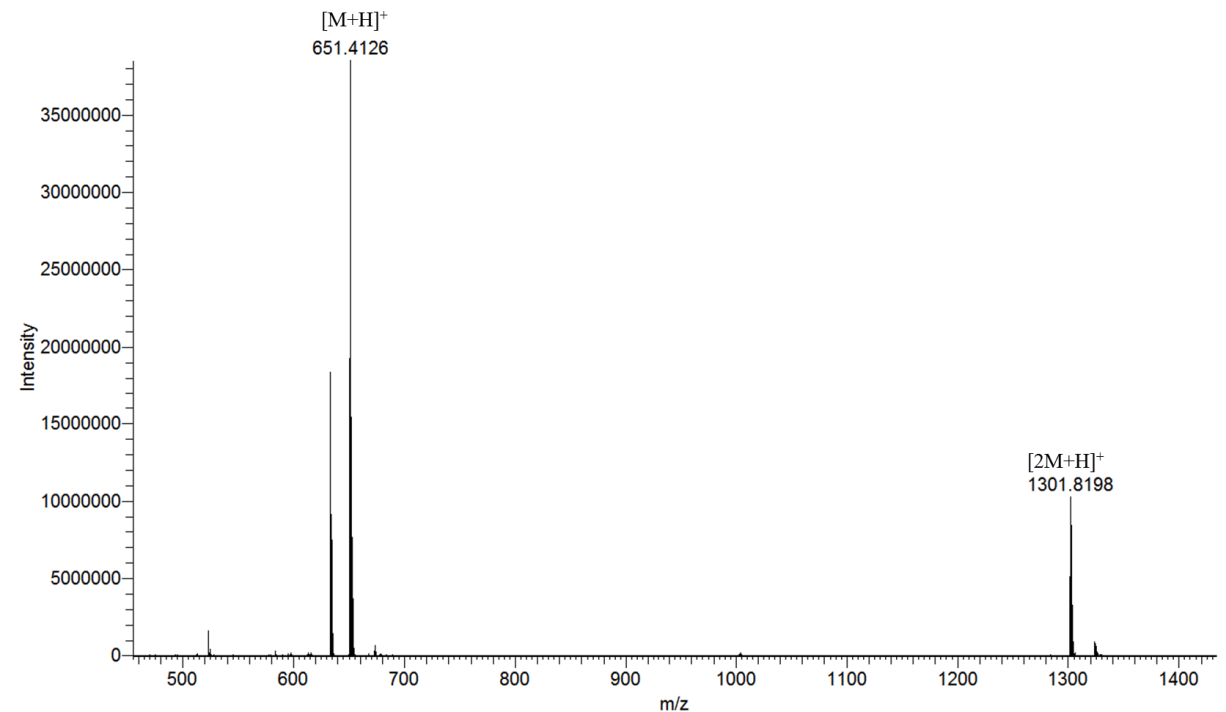


**Figure S4D.** The HRMS spectra of **4**.


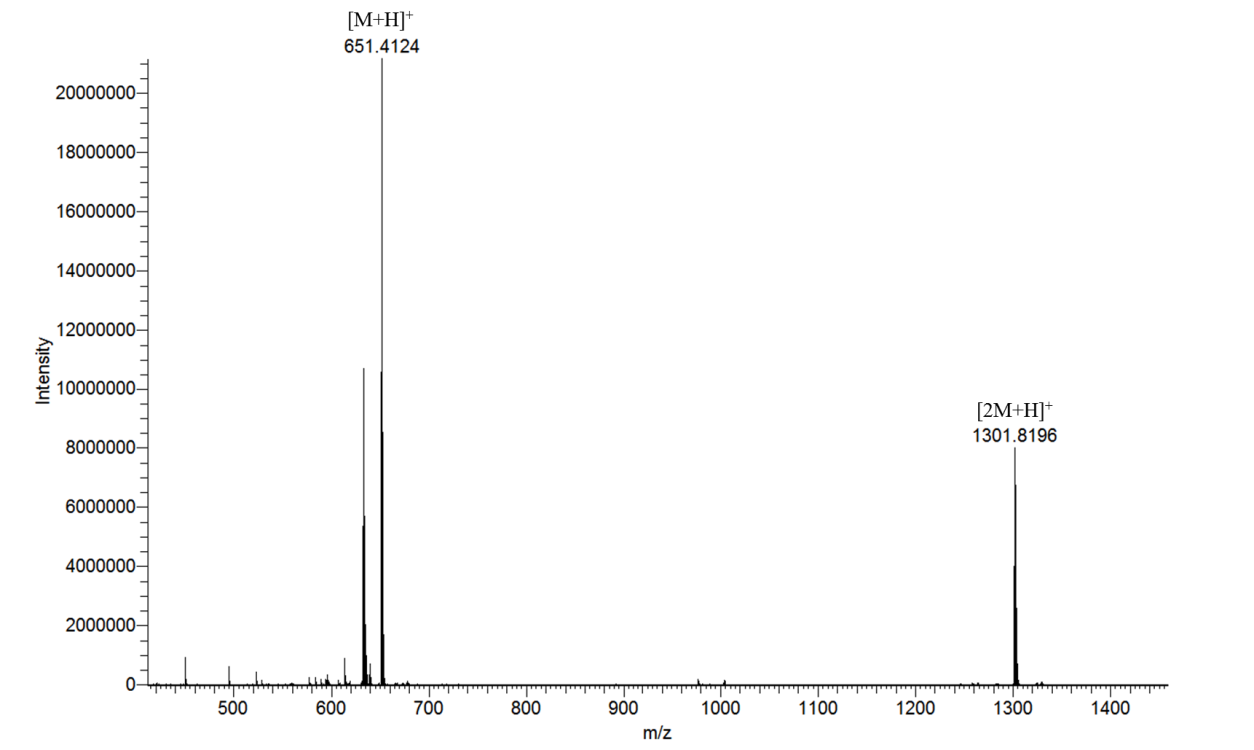


**Figure S4E.** The HRMS spectra of RDM A, **1**.

**(B)**

**(A)**

**Figure S5.** Inactivation of *orf(-2)*. (A) Construction of *orf(-2)* gene inactivation mutant. (B) PCR confirmation of the double-crossover mutant. M: DNA marker; W: *S. youssoufiensis* OUC6819 wild type strain; Mutation: *orf(-2)* gene inactivation mutant.

**(B)**

**(A)**

**Figure S6.** Inactivation of *orf(-1)*. (A) Construction of *orf(-1)* gene inactivation mutant. (B) PCR confirmation of the double-crossover mutant. M: DNA marker; W: *S. youssoufiensis* OUC6819 wild type strain; Mutation: *orf(-1)* gene inactivation mutant.

**(B)**

**(A)**

**Figure S7.** Inactivation of *orf1*. (A) Construction of *orf1* gene inactivation mutant. (B) PCR confirmation of the double-crossover mutant. M: DNA marker; W: *S. youssoufiensis* OUC6819 wild type strain; Mutation: *orf1* gene inactivation mutant.

**(A)**

**(B)**

**Figure S8.** Inactivation of *rdmA*. (A) Construction of *rdmA* gene inactivation mutant. (B) PCR confirmation of the double-crossover mutant. M: DNA marker; W: *S. youssoufiensis* OUC6819 wild type strain; Mutation: *rdmA* gene inactivation mutant.

**(B)**

**(A)**

**Figure S9.** Inactivation of *rdmB*. (A) Construction of *rdmB* gene inactivation mutant. (B) PCR confirmation of the double-crossover mutant. M: DNA marker; W: *S. youssoufiensis* OUC6819 wild type strain; Mutation: *rdmB* gene inactivation mutant.

**(B)**

**(A)**

**Figure S10.** Inactivation of *rdmC*. (A) Construction of *rdmC* gene inactivation mutant. (B) PCR confirmation of the double-crossover mutant. M: DNA marker; W: *S. youssoufiensis* OUC6819 wild type strain; Mutation: *rdmC* gene inactivation mutant.

**(B)**

**(A)**

**Figure S11.** Inactivation of *rdmD*. (A) Construction of *rdmD* gene inactivation mutant. (B) PCR confirmation of the double-crossover mutant. M: DNA marker; W: *S. youssoufiensis* OUC6819 wild type strain; Mutation: *rdmD* gene inactivation mutant.

**(B)**

**(A)**

**Figure S12.** Inactivation of *rdmE*. (A) Construction of *rdmE* gene inactivation mutant. (B) PCR confirmation of the double-crossover mutant. M: DNA marker; W: *S. youssoufiensis* OUC6819 wild type strain; Mutation: *rdmE* gene inactivation mutant.

**(B)**

**(A)**

**Figure S13.** Inactivation of *rdmF*. (A) Construction of *rdmF* gene inactivation mutant. (B) PCR confirmation of the double-crossover mutant. M: DNA marker; W: *S. youssoufiensis* OUC6819 wild type strain; Mutation: *rdmF* gene inactivation mutant.

**(B)**

**(A)**

**Figure S14.** Inactivation of *rdmL*. (A) Construction of *rdmL* gene inactivation mutant. (B) PCR confirmation of the double-crossover mutant. M: DNA marker; W: *S. youssoufiensis* OUC6819 wild type strain; Mutation: *rdmL* gene inactivation mutant.

**(B)**

**(A)**

**Figure S15.** Inactivation of *rdmM*. (A) Construction of *rdmM* gene inactivation mutant. (B) PCR confirmation of the double-crossover mutant. M: DNA marker; W: *S. youssoufiensis* OUC6819 wild type strain; Mutation: *rdmM* gene inactivation mutant.

**(B)**

**(A)**

**Figure S16.** Inactivation of *rdmO*. (A) Construction of *rdmO* gene inactivation mutant. (B) PCR confirmation of the double-crossover mutant. M: DNA marker; W: *S. youssoufiensis* OUC6819 wild type strain; Mutation: *rdmO* gene inactivation mutant.

**References**

1. Datsenko KA, Wanner BL. One-step inactivation of chromosomal genes in *Escherichia coli* K-12 using PCR products**.** Proc NatI Acad Sci. 2000; 97:6640-6645.

2. MacNeil DJ, Gewain KM, Ruby CL, Dezeny G, Gibbons PH, MacNeil T. Analysis of *Streptomyces avermitilis* genes required for avermectin biosynthesis utilizing a novel integration vector**.** Gene. 1992; 111:61-68.

3. Che Q, Li T, Liu X, Yao T, Li J, Gu Q, Li D, Li W, Zhu T. Genome scanning inspired isolation of reedsmycins A–F, polyene-polyol macrolides from *Streptomyces* sp. CHQ-64**.** RSC Advances. 2015; 5:22777-22782.

4. Gust B, Challis GL, Fowler K, Kieser T, Chater KF. PCR-targeted Streptomyces gene replacement identifies a protein domain needed for biosynthesis of the sesquiterpene soil odor geosmin**.** Proc NatI Acad Sci. 2003; 100:1541-1546.

5. Swick AG, Janicot M, Cheneval-Kastelic T, McLenithan JC, Lane MD. Promoter-cDNA-directed heterologous protein expression in *Xenopus laevis* oocytes**.** Proc NatI Acad Sci. 1992; 89:1812-1816.
